# Welcome to *Silence*

**DOI:** 10.1186/1758-907X-1-1

**Published:** 2010-01-12

**Authors:** David C Baulcombe, Phillip D Zamore

**Affiliations:** 1 Editors-in-Chief, Silence, BioMed Central, London, UK

## Editorial

Welcome to *Silence *[1], a new open-access journal devoted to RNA silencing and other pathways directed by non-coding RNAs. *Silence *springs from the extraordinary, yet brief, history of RNA silencing. In just two decades, we have seen the anomalous properties of plant and fungal transgenes connect with a series of amazing experiments in which injected double-stranded RNA triggered silencing in worms. These diverse lines of research revealed the essence of RNA interference (RNAi), and the importance of these discoveries has been recognized through numerous awards and accolades including a Nobel Prize for Fire and Mello [2,3].

Our current understanding of RNA silencing derives from experiments performed in organisms from three kingdoms, experiments that directly inspired billion-dollar investments by biotechnology and pharmaceutical companies to use RNA-silencing both to diagnose and to treat disease in humans. Both small interfering RNAs and microRNA-blocking antisense oligonucleotides are now in human clinical trials [4]. Some of the first GM plants to be produced exploited RNA silencing, although the mechanisms were not well understood at the time [5,6]. The study of RNA silencing produced enabling technology that allows each gene in a sequenced genome--even cultured human cells--to be knocked out or knocked down, providing a lifeline to functional genomics. There can be no question that RNA silencing research has had an impact!

RNA silencing has excited scientists and non-scientists alike: witness front-page headlines in the American and British press [7-10], even before the Nobel Prize. Such interest, of course, reflects the power of RNA silencing as biotechnology. But equally important is that RNA silencing exemplifies the elegant creativity of natural selection. Just as we might gaze in awe at a blue whale in the ocean (how can such a creature exist?), we marvel at the simple principles and complex molecular machines that underlie RNA silencing pathways. The role of silencing as an antiviral defence in plants and invertebrates illustrates this point: it uses the sequence of the invading virus itself to define the targets to be repressed and so has infinite specificity [11]. As a defense system RNA silencing is unsurpassed.

The study of RNA silencing has now travelled far from its posttranscriptional roots. The link between RNA and epigenetic silencing by chromatin modification, for example, is well established in many organisms [12]. In other developments the discovery of novel families of small silencing RNAs continues to expand the universe of guides far beyond the original microRNA and small interfering RNA pioneers [13]. This diversity is not mere molecular icing on the RNAi cake, because silencing underpins biological phenomena as diverse as virus resistance, control of chromosome architecture, transposon activity, genome rearrangement, and development, as well as responses to biotic and abiotic stimuli [14].

In parallel, other types of RNA-mediated mechanisms have been discovered, from CRISP RNAs [15] in bacteria to unexpectedly large families of non-coding RNAs derived from the intergenic regions of animals and plants [16]. These discoveries have been informed by, and in turn enrich the intellectual framework of RNAi. Thus, *Silence *will enthusiastically publish papers on these and other RNA-based mechanisms in addition to studies of the canonical RNA silencing pathways.

Papers with (RNA) AND (silence OR silencing) in their titles or abstracts first appeared in the mid 1990s; there are now more than 1,400 each year and the trend is increasing (source: Web of Science) [17]. So why introduce a new journal if these papers are already finding a home? Two answers explain our motivation in founding *Silence*. First, the history of silencing is one of extensive cross-fertilization among different research communities. Such inter-organism as well as inter-disciplinary collaboration and discussion explains the remarkable productivity of our field. Unfortunately, the expansion and diversification of RNA silencing research threatens to fragment our intellectual community. Increasingly, the opportunity for a plant researcher, for example, to read a paper on genomic rearrangements in protozoa or for a fly geneticist to learn of the discovery of a novel mechanism of transposon control in fungi is being lost. Of course, existing journals devoted to the study of RNA will always publish some RNA silencing papers, but it is unreasonable to think that these journals can allocate a high proportion of their pages to our field. As RNA silencing research diversifies, we risk losing the excitement generated by the common enthusiasm for RNA silencing that unites distinct research communities. *Silence *seeks to nurture that enthusiasm by sustaining the interdisciplinary flavor of our field.

Second, *Silence *is a response to the rise of genomics and high throughput sequencing. These developments challenge molecular biologists because they demand a new, computational outlook on biological data. There is an enormous opportunity for bioinformaticians, mathematicians and statisticians to work together with experimental biologists to meet this challenge. They will extract useful and interesting information from genome sequences and large datasets and integrate them with similarly large datasets dealing with various other "omic" analyses of experimental systems. Modeling as a basis for hypothesis generation and testing will become increasingly important.

*Silence *can help molecular biologists and geneticists communicate effectively with computational scientists. We would be pleased, for example, to publish computational tools and research papers that use "dry science" to investigate RNA silencing or non-coding RNAs. We welcome reviews and commentaries in which computationalists introduce novel ideas, approaches and concepts in a style that is accessible to experimentalists.

This inaugural issue of *Silence *presents a selection of articles on different topics and an insightful review to illustrate the type of paper that we would like to include as the journal grows. Our renowned and diverse editorial advisory board [18] ensures fair but rigorous peer review, and our open access publication pipeline provides an efficient and easy-to-use system run by the well established BMC team. All BioMed Central journals are included in PubMed Central [19] and other freely accessible full-text repositories. This complies with the open access policies [20] of many funders including those of the Howard Hughes Medical Institute, NIH, and Wellcome Trust [21-23].

We look forward to receiving your manuscripts for publication and your feedback about the journal. *Silence *is meant to be yours; your comments and submissions will ensure it succeeds as the hub of the RNA silencing field.
